# Trends in consultations and prescribing for rheumatic and musculoskeletal diseases: an electronic primary care records study

**DOI:** 10.3399/BJGP.2022.0648

**Published:** 2023-09-19

**Authors:** Victoria K Welsh, Kayleigh J Mason, James Bailey, Ram Bajpai, Kelvin P Jordan, Christian D Mallen, Claire Burton

**Affiliations:** Centre for Musculoskeletal Health Research, School of Medicine, Keele University, Keele.; Centre for Musculoskeletal Health Research, School of Medicine, Keele University, Keele.; Centre for Musculoskeletal Health Research, School of Medicine, Keele University, Keele.; Centre for Musculoskeletal Health Research, School of Medicine, Keele University, Keele.; Centre for Musculoskeletal Health Research, School of Medicine, Keele University, Keele.; Centre for Musculoskeletal Health Research, School of Medicine, Keele University, Keele.; Centre for Musculoskeletal Health Research, School of Medicine, Keele University, Keele.

**Keywords:** analgesia, COVID-19 pandemic, drug prescribing, musculoskeletal diseases, musculoskeletal pain, primary care

## Abstract

**Background:**

Rheumatic and musculoskeletal diseases (RMDs) are common and generally managed in primary care through supported self-care, physiotherapy, analgesia, and specialist referral where indicated. The COVID-19 pandemic led to abrupt changes in primary care delivery, including moves to remote consulting, pauses on group-based self-care, and restricted referrals.

**Aim:**

To describe how patterns of UK primary healthcare consultations and analgesic prescribing relating to RMDs changed during the COVID-19 pandemic.

**Design and setting:**

Observational study using routinely collected national primary care electronic health record data from the Clinical Practice Research Datalink between 1 April 2017 and 1 October 2021.

**Method:**

RMD and analgesic SNOMED-CT codes were derived through consensus and published work. Prevalent and incident RMD-related consultations were determined, and RMD consultations matched to prevalent and incident analgesia prescriptions. Joinpoint regression was used to describe trends over time.

**Results:**

Prevalent and incident RMD consultations steadily increased until March 2020 when a substantial drop occurred as pandemic- related restrictions were introduced; levels had not recovered to pre-pandemic highs by October 2021. While incident and prevalent analgesic prescribing also reduced around March 2020, the proportion of patients with an RMD consultation prescribed any analgesic increased from 27.72% in February 2020 to 38.15% in April 2020, with increases across all analgesic groups. A higher proportion of strong opioid prescriptions was seen in the most deprived areas.

**Conclusion:**

Pandemic-associated restrictions led to fewer primary care consultations and relative increases in analgesic prescribing, including strong opioids, for RMDs in the UK. Policymakers must consider the impact of these changes in future healthcare resource planning.

## INTRODUCTION

Rheumatic and musculoskeletal diseases (RMDs), defined as problems of the joints, muscles, and bones,^1^ cause a high disease burden globally.^2^ In the UK, primary care is generally the first point of care for adults and children with RMDs, with 20% of adults consulting for an RMD annually.^3^ A multidisciplinary approach to management is recommended including advice, self- care, and referral for non-pharmacological treatments such as physiotherapy, exercise, and weight management. Analgesia is also often prescribed to relieve symptoms, although harmful drug-related adverse events can occur, particularly in older adults.^4^^,^^5^ In March 2020, the COVID-19 pandemic prompted UK primary care delivery to change abruptly.^6^ The ‘total-triage’ care model, whereby all consultations were ‘remote- by- default’, was widely implemented,^7^^,^^8^ replacing traditional face-to-face health care. The government issued a ‘stay at home’ order and instigated a ‘lockdown’.^9^ Primary care was encouraged to reduce routine referrals to community and secondary care to increase acute care capacity, including to services that support non-pharmacological management of RMDs.^10^^,^^11^

These restrictions resulted in an approximately 30% reduction in primary care consultations per person, which persisted until June 2020 before returning to pre-pandemic levels in September 2020.^6^ To the authors’ knowledge, there are no published studies addressing changes in primary healthcare services for people living with RMDs during the first 18 months of the pandemic. The aim of this study, therefore, was to describe patterns of RMD-related primary care consultations and prescribing immediately before, during, and after UK pandemic-related restrictions, addressing the hypotheses that there would be a reduction in consultations and an increase in analgesic prescribing, both in volume and strength, to manage RMDs.

## METHOD

### Setting, patients, and timeline

Longitudinal routinely collected electronic primary care record data from the Clinical Practice Research Datalink (CPRD) Aurum database were analysed from 1 April 2017 to 1 October 2021. No exclusion criteria were applied. CPRD Aurum collates anonymised routinely recorded primary care data of >40 million patients, including 15 million currently registered patients (over 20% of the UK population) from >1500 general practices using EMIS Web software. Aurum includes practices from England (99% of Aurum) and Northern Ireland (1%) only. Aurum is representative of the English population on age, sex, deprivation, and geographical spread.^12^ Linked data were obtained for patient-level Index of Multiple Deprivation. National lockdown periods in England were as follows: lockdown 1 (23 March 2020 to 23 June 2020), lockdown 2 (5 November 2020 to 2 December 2020), and lockdown 3 (6 January 2021 to 29 March 2021).^13^ Lockdown periods were similar for Northern Ireland.

**Table:** How this fits in

Rheumatic and musculoskeletal disorders (RMDs) are a common cause of pain and disability, with core non-pharmacological management supported by analgesic medications. To the authors’ knowledge, no previous studies have observed the impact of the COVID-19 pandemic on the care of patients with RMDs in primary care, including consultation patterns and analgesic prescribing. The results of this study show that fewer patients consulted with RMDs during lockdown, and a greater proportion of those who consulted were prescribed strong analgesia (including opioids) during pandemic-related restrictions. Clinicians appeared to respond to patients’ needs during the pandemic amid restrictions placed on non-pharmacological treatments, and commissioners must consider the impact of these behaviour changes during future pandemic planning.

### Outcomes

Study outcomes were prevalence and incidence of primary care consultations for RMDs and associated analgesic prescribing. RMD consultation codes were based on previous published work^3^ and updated with relevant SNOMED-CT codes. All included codes were assigned a label of non- inflammatory RMD, inflammatory RMD, traumatic injury, or neoplastic. Each code was further assigned a pain phenotype (osteoporosis; chronic, multi-site, or generalised pain; trauma; malignancy; inflammatory condition; referral to specialist; or other) and site-specific region (hip/pelvis; knee; spine; axial; distal lower limb; proximal upper limb; or distal upper limb). Code lists and groups were developed through a consensus exercise between two GPs and two consultant rheumatologists.

Analgesic prescriptions were categorised according to previous published research,^14^ with the addition of duloxetine.^15^ All prescriptions in the British National Formulary (BNF) chapters for opioid analgesics, non- opioid analgesics, and non- steroidal anti-inflammatory drug (NSAID) medications (BNF 4.7.1, 4.7.2, 10.1.1.1, and 10.3.2)^16^ were included and combined with gabapentinoids and duloxetine. Each code was grouped into 1) basic analgesics, 2) weak analgesics: weak combination opioids, 3) moderate analgesics: moderate combination opioids, 4) strong analgesics: strong combination opioids and opioids, 5) very strong analgesics, 6) NSAIDs, 7) neuropathic agents including amitriptyline and gabapentinoids, or 8) serotonin noradrenaline reuptake inhibitors (SNRIs) (duloxetine). For code lists, see https://www.keele.ac.uk/mrr/codelists/musculoskeletalcodelists.

### Data analysis

Monthly RMD consultation prevalence and incidence rates per 10 000 registered persons were calculated by sex, age band (0–4, 5–9, 10–14, 15–24, 25–34, 35–44, 45–54, 55–64, 65–74, 75–84, and ≥85 years), and geographical region (England only: East Midlands, East of England, London, North East, North West, South Central, South East Coast, South West, West Midlands, and Yorkshire and the Humber). RMD consultation prevalence and incidence were determined overall, by pain phenotype, and pain region.

Monthly prevalence and incidence rates of analgesic prescribing (using groups 1–8) on the same date as an RMD consultation per 10 000 persons (95% confidence interval [CI]) were calculated by sex and age bands.

RMD prevalence was defined as the total number of patients with at least one coded consultation of RMD within each monthly period divided by the total registered database population at the start date of that period and expressed per 10 000 persons. Incident RMD consultations were defined (as in a previous study)^17^ by the total number of patients with at least one coded consultation for any RMD within each monthly period who had no recorded RMD of any type in the previous 24 months divided by the total registered database population without a RMD consultation at the start date of the period and expressed per 10 000 persons.

Prevalent prescribing was defined as the total number of patients prescribed an analgesic category in each monthly period divided by the total registered database population at the start date of the period and expressed per 10 000 persons, and includes repeat prescribing. Incident prescribing was defined as the total number of patients prescribed an analgesic in each monthly period without any prescription from the same analgesic group in the previous 24 months divided by the total registered database population without a previous analgesic prescription at the start date of the period and expressed per 10 000 persons, and includes new prescribing for pre-existing and new RMDs.

Analgesic prescribing was also presented as the proportion of persons with a prevalent or incident prescription on the day of RMD consultation in each calendar month. The Index of Multiple Deprivation^18^ was available for the numerator population to allow analysis by deprivation quintiles.

Joinpoint regression was used to determine months (joinpoints) where there were significant changes in trends over time, with mean monthly percentage change (MPC) and 95% CI determined for each time period between these joinpoints. When interpreting joinpoint regression, a positive value MPC suggests an increasing trend and a negative value suggests a decreasing trend*.* The optimal number of joinpoints (up to a maximum of five) were selected using a permutation test, which determines significant changes in trends, with 4500 permutations and a significance level of *P*<0.05. Joinpoint analyses were performed using Joinpoint software (version 4.9.0.0).

## RESULTS

The numerator population (individuals presenting with an RMD-related primary care consultation) included 6 057 747 patients from practices in England and Northern Ireland. The denominator population remained largely constant over the study period at approximately 13.5 million individuals.

### RMD prevalence and incidence

**Figure 1.**
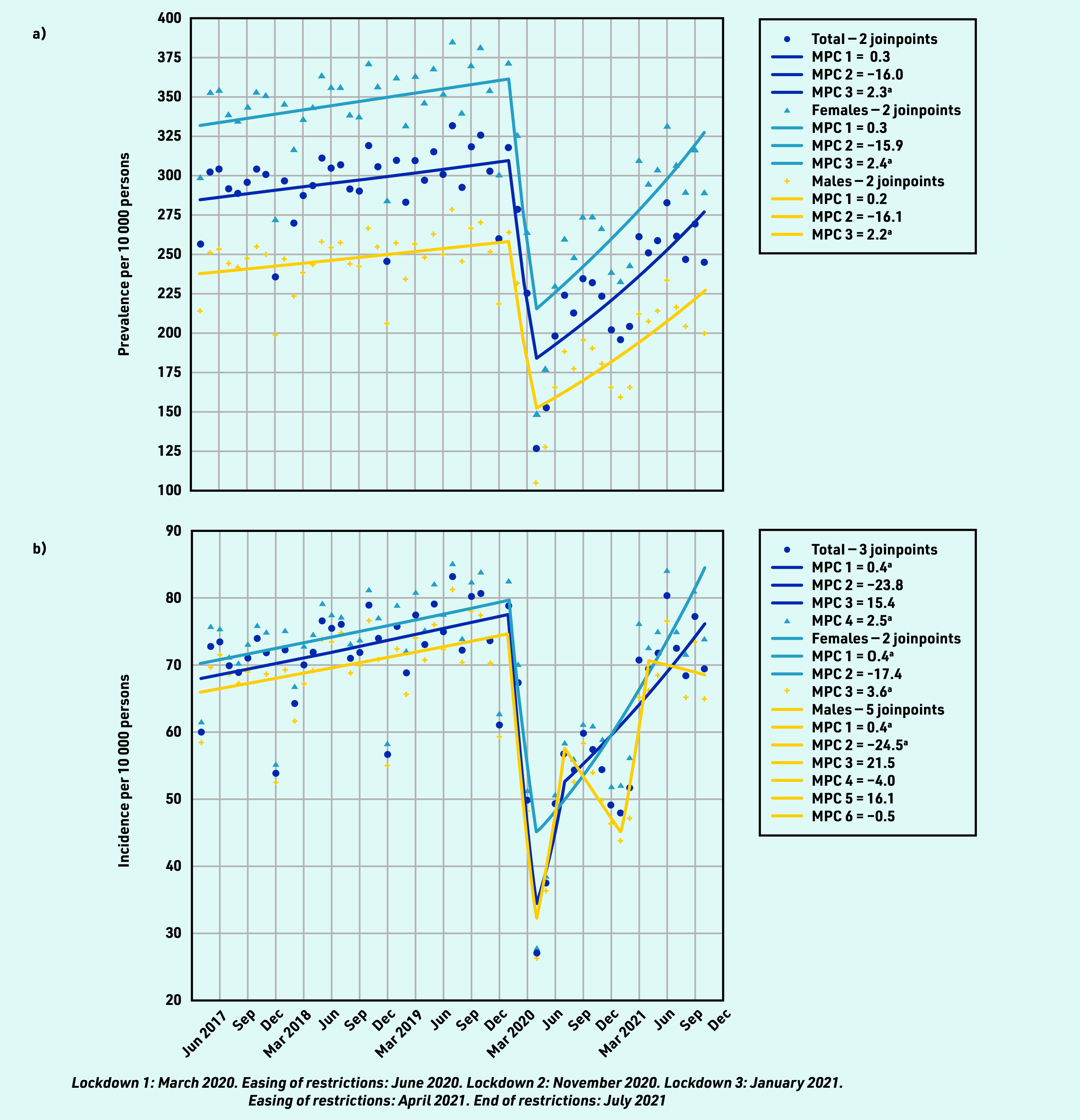
*Monthly a) prevalent and b) incident consultations for rheumatic and musculoskeletal diseases per 10 000 persons between April 2017 and October 2021.* *^a^The MPC is significantly different from zero at alpha = 0.05. MPC = mean monthly percentage change.*

The monthly prevalence of individuals consulting with RMDs increased gradually between April 2017 and January 2020 by a mean MPC of 0.3%, from 256.53 (95% CI = 255.67 to 257.39) to 317.86 (95% CI = 316.92 to 318.80) per 10 000 persons (Figure 1 and Supplementary Table S1). Between February 2020 and April 2020, there was an MPC of −16.0% in prevalent consulting individuals to 225.39 (95% CI = 224.59 to 226.19) in March and 126.56 (95% CI = 125.95 to 127.17) per 10 000 persons in April 2020. From May 2020 to October 2021 there was an increase of MPC to 2.3% in the number of prevalent RMD consulters; however, the prevalence rate did not recover to pre- pandemic levels as of October 2021 when 244.66 (95% CI = 243.82 to 245.50) per 10 000 persons consulted with an RMD.

Trends in RMD prevalence were similar across sex, age, geographical region, and phenotype (see Supplementary Tables S1 and S2 for details), except for the youngest age groups (0–4, 5–9, 10–14, and 15–24 years) where consultation rates were declining from July 2019 into the pandemic, and for ‘inflammatory arthritis’ (including rheumatoid arthritis and juvenile inflammatory arthritis), where consultation prevalence did not demonstrate an equivalent substantial reduction.

RMD incidence (Figure 1) followed similar patterns as observed for prevalence, except for males, whose incident presentations demonstrated seasonal variation (see Supplementary Tables S3 and S4, and Supplementary Figure S1 for details).

### Analgesic prescribing: prevalence and incidence

**Figure 2.**
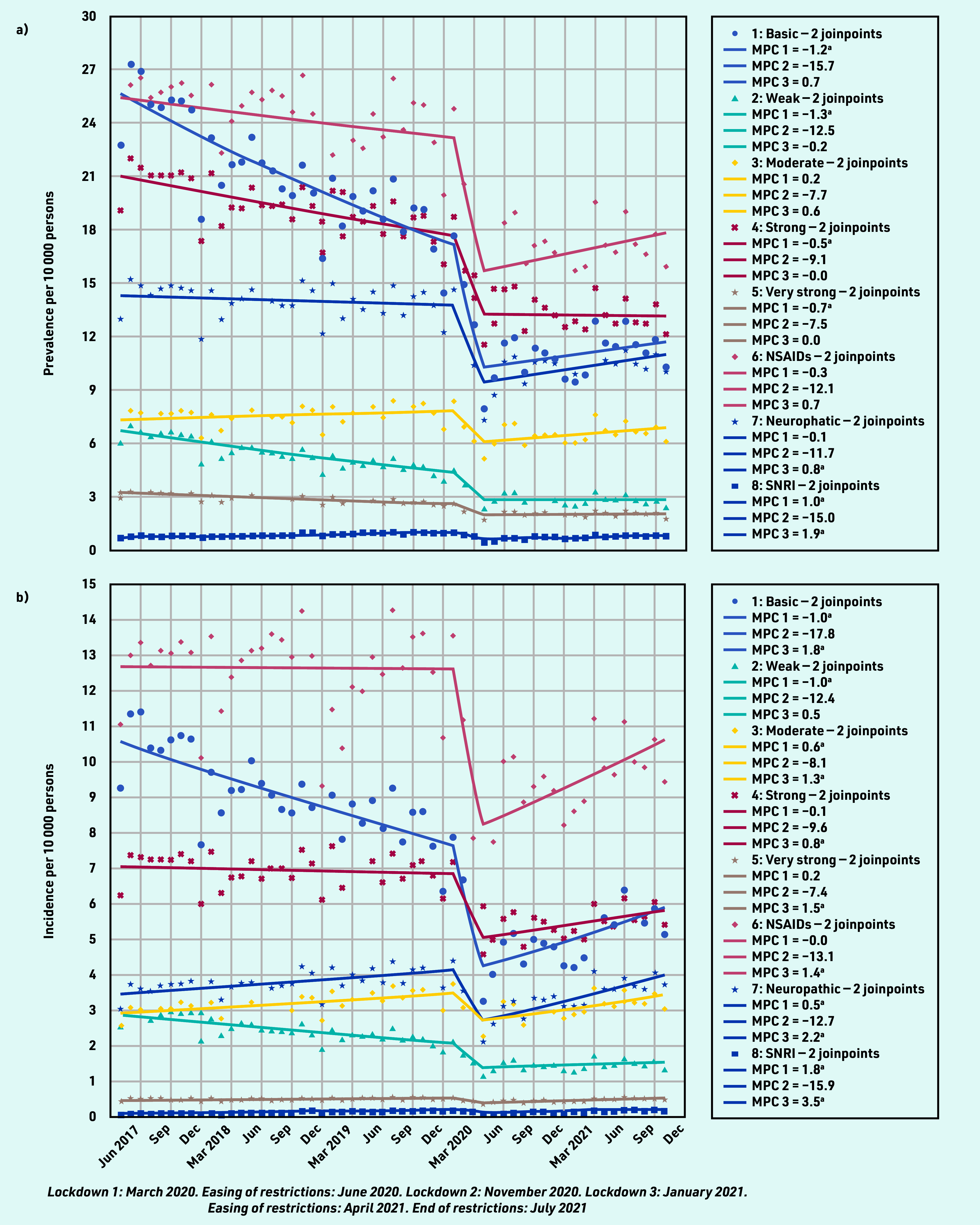
*Total a) prevalent and b) incident analgesic prescribing on the day of a consultation for rheumatic and musculoskeletal diseases.* *^a^ The MPC is significantly different from zero at alpha = 0.05. MPC = mean monthly percentage change. NSAID = non-steroidal anti-inflammatory drug. SNRI = serotonin noradrenaline reuptake inhibitors.*

Prevalent analgesic prescribing varied by analgesic type. Between April 2017 and January 2020, prescribing of basic analgesics (MPC −1.2%, 95% CI = −1.5% to −0.9%); weak (MPC −1.3%, 95% CI = −1.5% to −1.0%), strong (MPC −0.5%, 95% CI = −0.7% to −0.3%), and very strong opioids (MPC −0.7%, 95% CI = −0.9% to −0.5%); NSAIDs (MPC −0.3%, 95% CI = −0.6% to −0.0%); and neuropathic analgesics (MPC −0.1%, 95% CI = −0.3% to 0.1%) fell gradually while prescribing of moderate opioids (MPC 0.2%, 95% CI = 0.0% to 0.4%) and SNRIs increased slightly (MPC 1.0%, 95% CI = 0.7% to 1.2%) (Figure 2 and Supplementary Table S1). Between February and April 2020, prescribing of all analgesics fell at a greater rate with the largest relative change for NSAIDs (MPC −12.1%, 95% CI = −32.6% to 14.6%), basic analgesics (MPC −15.7%, 95% CI = −35.5% to 10.1%), and neuropathic analgesics (MPC −11.7, 95% CI = −27.2% to 7.1%). From May 2020 to October 2021, prescribing of analgesia remained much lower than pre-pandemic, with only modest relative increases in basic analgesics (MPC 0.7%, 95% CI = −0.2% to 1.7%), moderate opioids (MPC 0.6%, 95% CI = 0.0% to 1.3%), NSAIDs (MPC 0.7%, 95% CI = −0.2% to 1.6%), and neuropathics (MPC 0.8%, 95% CI = 0.2% to 1.5%). SNRIs demonstrated the greatest relative increase in prescribing (MPC 1.9%, 95% CI = 1.1% to 2.6%).

Incident analgesic prescribing showed more variable patterns (Figure 2 and Supplementary Table S3). NSAID prescribing remained constant until February 2020 when prescribing reduced, then increased from May 2020 until the end of the study period in October 2021. Prescriptions of basic, weak, and strong analgesics were decreasing before the pandemic and continued to reduce, but at a greater rate, through the early months of the pandemic. Prescriptions for moderate and very strong analgesics were increasing in the pre-pandemic period, decreased in line with other analgesia from February 2020, and returned to an increasing trend in May 2020. New prescriptions for neuropathic analgesics increased through February 2020 where they declined initially and then increased again from May 2020. SNRI prescribing gradually increased until February 2020 when it reduced, then rose slightly from May 2020.

### Prevalent and incident prescribing as a proportion of consultation episodes

**Figure 3.**
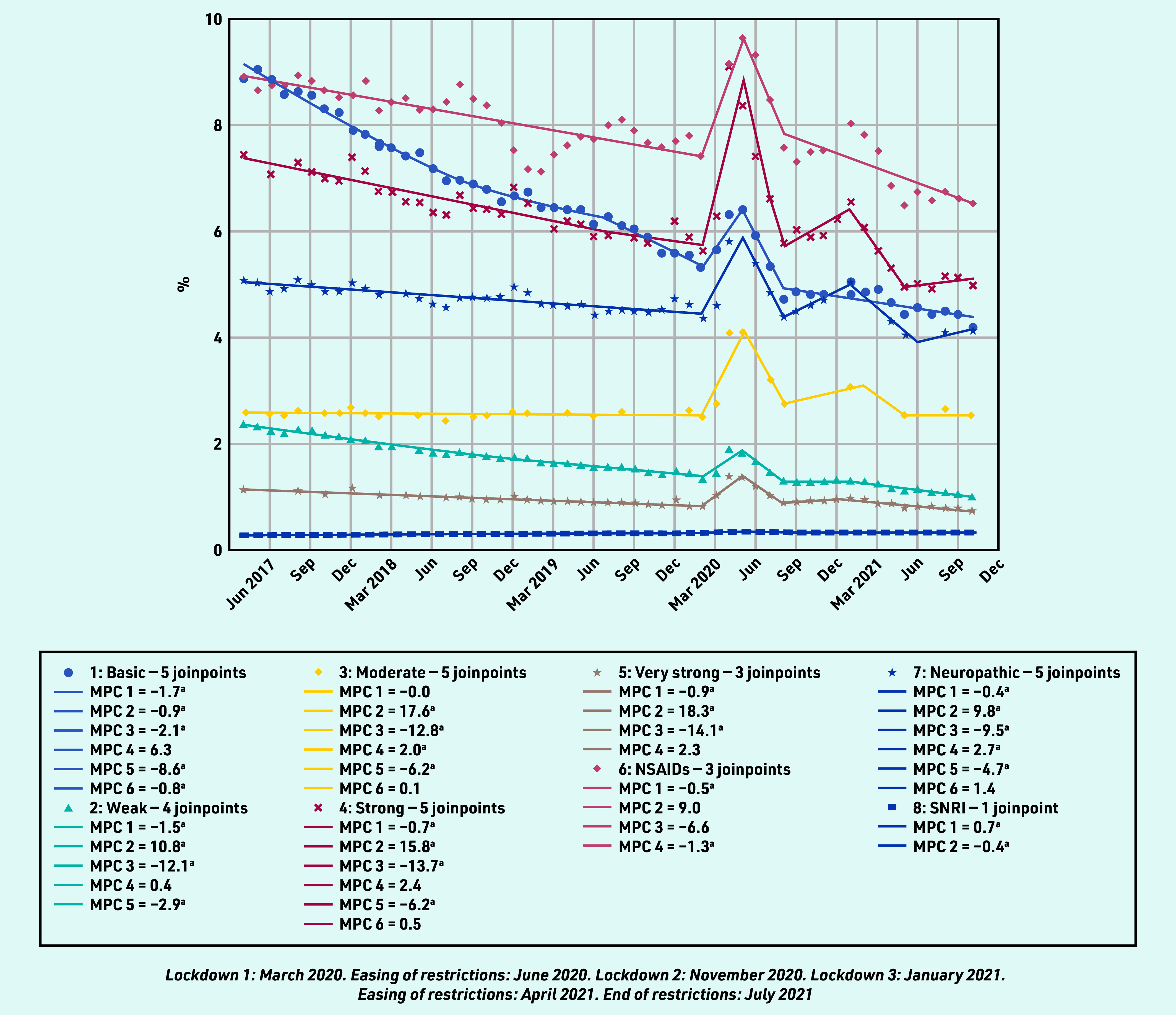
*Proportion of prevalent prescribing on the day of a consultation for rheumatic and musculoskeletal diseases.* *^a^The MPC is significantly different from zero at alpha = 0.05. MPC = mean monthly percentage change. NSAID = non-steroidal anti-inflammatory drug. SNRI = serotonin noradrenaline reuptake inhibitors.*

**Figure 4.**
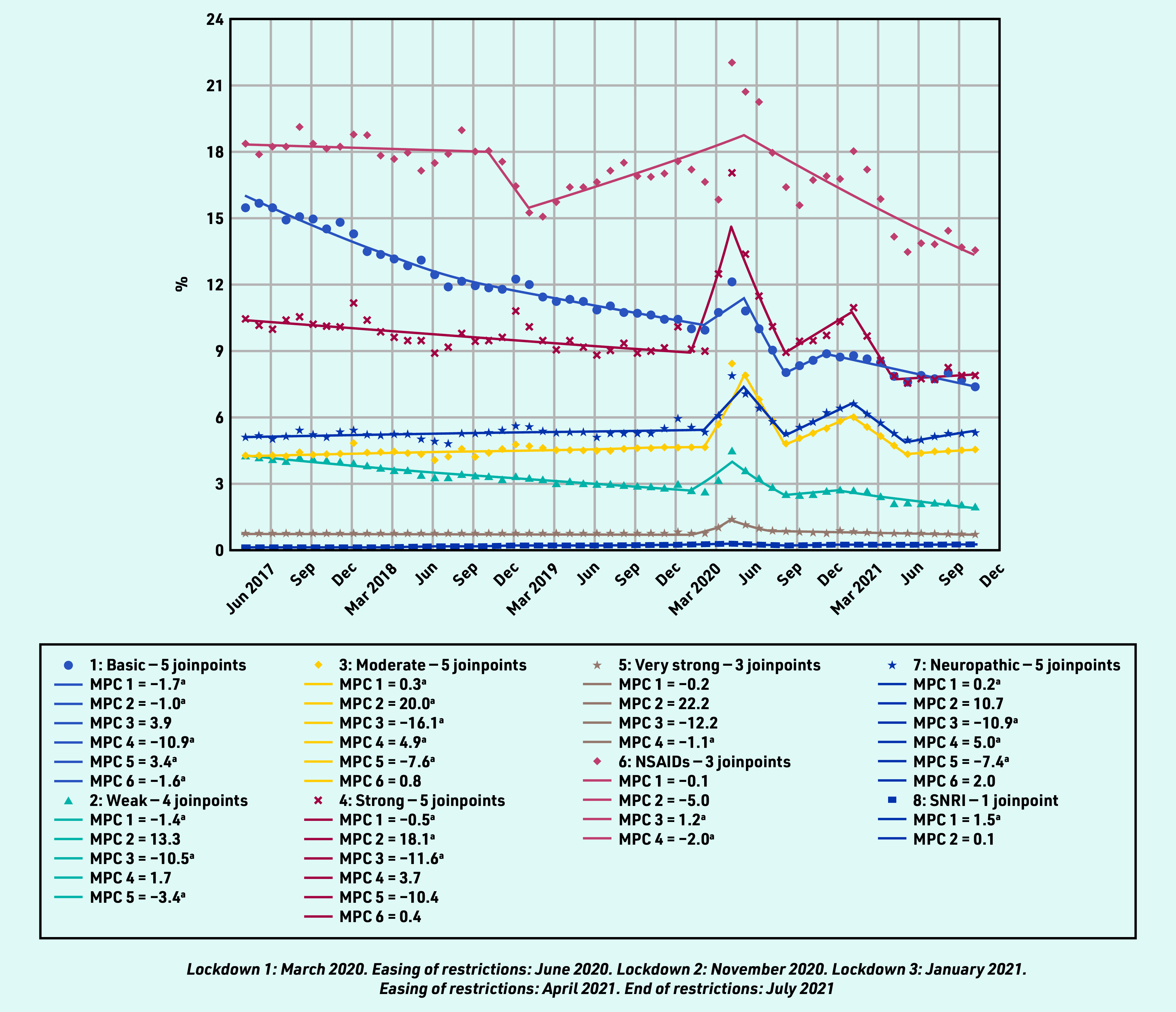
*Proportion of incident analgesic prescribing on the day of a consultation for rheumatic and musculoskeletal diseases.* *^a^The MPC is significantly different from zero at alpha = 0.05. MPC = mean monthly percentage change. NSAID = non- steroidal anti- inflammatory drug. SNRI = serotonin noradrenaline reuptake inhibitors.*

In contrast to the pattern of reduced overall analgesia prescribing in the pre- pandemic period, the percentage of all RMD consultations with either a prevalent or incident (first) analgesic prescription increased in all analgesic groups between March and May 2020, except for incident prescribing for NSAIDs, which had been increasing since February 2019, and SNRIs, which had been increasing since April 2017 (Figures 3 and 4), for example, the proportion of patients with an RMD consultation prescribed any analgesic increased from 27.72% in February 2020 to 38.15% in April 2020. This increase was evident for all analgesic groups, except SNRIs, which remained relatively static. The upsurge in prevalent analgesic prescribing was greatest for very strong opioid analgesics (MPC 18.3%), and this category was the second most prescribed analgesic to NSAIDs from April 2020 until the end of the study. As a proportion of those consulting, the overall prevalent and incident prescribing of simple analgesics, NSAIDs, and weak opioids continued in a downward trend after the first lockdown, whereas the prescribing of strong opioids, neuropathic analgesia, and moderate analgesia showed a further peak in prevalent and incident prescribing in the third lockdown period (January 2021).

Patients residing in more deprived areas were prescribed, relative to consultations, more basic, weak, and strong analgesia as a new prescription than those from less deprived areas (Supplementary Table S5 and Supplementary Figure S2). Prescribing patterns throughout the pandemic also appeared different in more deprived groups, with a discernible peak in prescribing as a proportion of consultations during the third lockdown in January 2021.

Analgesic prescribing varied substantially by age (see Supplementary Table S6 and Supplementary Figure S3). Those aged 25–64 years had the greatest increases in strong opioid prescribing around March 2020 compared with the increased prescribing of other analgesic groups; increased prescribing of all analgesic groups also occurred in the ≥75 years age group in March 2020, except for NSAIDs and neuropathics for adults aged ≥85 years.

## DISCUSSION

### Summary

Prevalent and incident RMD consultations were steadily increasing before the UK introduced pandemic-related restrictions in March 2020. A substantial drop in RMD consultations followed, which had not recovered to pre-pandemic levels by October 2021.

Analgesic prescribing for patients presenting with RMDs varied according to analgesic class, age, and deprivation status. Overall, there was a general decline in RMD- associated analgesic prescribing from early 2020, with an upturn from April 2020 for all analgesic categories, although not to pre- pandemic levels. Conversely, the proportion of RMD-associated consultations in which an analgesic was prescribed increased in March 2020, with notable upsurges in all analgesic groups, particularly strong and very strong opioid analgesics. Increases in prescribing were seen again during the second UK lockdown in moderate and strong opioids, and neuropathic medications.

### Strengths and limitations

This study used primary care data from an extensive registry of general practices across England and Northern Ireland, which is broadly representative of the total population,^12^ although medications that are available over the counter (including paracetamol, aspirin, ibuprofen, co-codamol 8/500, and rubefacients) and real-life analgesic use are not measured, leading to potential prescribing under- or overestimates. New guidance relating to chronic pain was published by the National Institute for Health and Care Excellence in April 2021.^19^ This guidance advises against the prescribing of paracetamol, NSAIDs, opioids, and gabapentinoids for chronic pain, and recommends the prescribing of antidepressant medication (including duloxetine and amitriptyline) if necessary. There are no corresponding joinpoints in the data to represent a change in habit associated with the introduction of this guidance, although this may be because the chronic pain phenotype represented a small proportion of patients consulting for RMDs. Complete ethnicity data were unavailable, and it is possible that consultation and prescribing trends vary according to ethnicity given that people from ethnic minority groups report poorer health outcomes despite controlling for socioeconomic disadvantages.^20^

### Comparison with existing literature

Increases in prescribing as a proportion of RMD consultations may have occurred because of a lack of available non- pharmacological management options during lockdown. It may be that only patients who were experiencing severe RMD symptoms presented to primary care and thus a proportion of consultations resulted in the prescribing of more, and stronger, analgesia. It is known that low levels of psychological distress are associated with the reporting of no pain;^21^ it may be that the observed increase in psychological distress through the pandemic period^22^ heightened people’s pain experiences and led to corresponding increases in analgesic prescribing as a proportion of RMD consultations. This study shows that trends in prescribing of analgesia in England were stable or slightly decreasing from 2017 up to the first lockdown. Previous studies have shown varied trends, including increases in prescribing of long-term opioids up to 2011 before slightly falling to 2013,^23^ an increase in codeine prescribing up to 2017,^24^ and an increase in prescribing of strong opioids in Wales between 2005 and 2015.^25^ The current study also supports a previous research finding that deprivation was strongly linked to opioid prescribing.^26^

### Implications for research and practice

UK-based restrictions associated with the COVID-19 pandemic have impacted on the care of people with RMDs in the community, with a short-term reduction in consultations and an increase in prescribing of strong analgesia with potentially harmful consequences. Policymakers and commissioners must take account of this when considering future restrictions and contemplate protecting non- pharmacological managements of pain, including physiotherapy and exercise. National strategies to address health inequalities^27^ and improve mental health^28^ are urged to consider RMDs and prescribing as part of the holistic approach to improve the health and wellbeing of the population. Further research is required to understand the impact of reduced consulting and increased analgesic prescribing during the early pandemic period, particularly for patients started on strong analgesia during lockdown to investigate subsequent repeat prescribing and primary care use as NHS services continue to see long waiting lists for non-urgent treatments.

## Data

Data may be obtained from a third party and are not publicly available. The data were obtained from the CPRD. CPRD data governance does not allow the authors to distribute patient data to other parties. Researchers may apply for data access at http://www.CPRD.com.
